# Cornell on Epilepsy

**Published:** 1850-09

**Authors:** W. M. Cornell

**Affiliations:** Boston, Mass.


### ARTICLE X.

*Too Early and Severe Mental Effort the Cause of Epilepsy in Children.*---By W. M. Cornell, M. D., of Boston, Mass.

There is often a wish among parents---perhaps a meritorious one,---to have their children bright and forward. The opening buds in the speaking countenance of the infant, as mental developement proceeds, are a source of the deepest interest. But it has been well said that "precocious children rarely make eminent men."

I protest against that system of education which attempts to make men and women, scholars and philosophers, of little children. This, in many places, seemed to be the design of the infant school system, so much in vogue a few years past. We were upon the eve of having *learned* children, of the age of five or six years: and the sage fathers, and good mothers, and maiden aunts, and teachers, seemed about to astonish the world by their prodigies. Many looked on with astonishment, and began to wonder what kind of men and women we were to have from *such* children. But they were soon relieved of their difficulty, for it proved that such "forward" children never, or rarely, if ever, lived to be men and women.

The mind of the child, which was urged forward thus prematurely, soon became jaded and lost its balance. Nervous disease soon supervened, and the pretty, little, bright and sprightly child, the idol of its fond parents, their "little pride," soon became the object of their solicitude, and deep and painful anxiety. The precocious intellect that even in youth could understand and read English, Latin, and French, to the admiration of all, so affected the body, that wonderful mechanism, as to overstretch those delicate strings, the nerves, and induce that worst and most to be dreaded of all irritations, *nervous*. The nervous excitement increased, and the bodily health began to fail. The bright flashes of thought, the sparkling witicisms of the pale and feeble little being, bursting from the overtasked intellect, called forth the loudest applause from inconsiderate friends and ignorant admirers.

The scene soon changed. The nervous spasms began.---The body, as the mind and nervous system were thus overburdened, craved more food as it was able to bear less. The twitchings and spasms increased, till by some extra mental effort, or sudden fright, or overloaded stomach, the spasm became the convulsion, and Epilepsy, with all its horrors, was apparent; and, in all probability, irretrievable *idiocy*, to cap this climax of a wrong education, lies before this innocent child.

This is only one of the forms in which this monstrous perversion of the laws of our being, manifests itself, and I have taken this view of it from the fact that it has been often brought before me, in all its horrors, during the last few years. Among the numerous causes of Epilepsy which have come under my notice, this has been a prolific one: and, I warn that fond but ambitious parent, who is anxious to see his child a genius, to beware of his course. He is planting a tree, which instead of furnishing him with a refreshing shade in his old age, will throw the poison of the bitterest reflection over the meridian of his life, in the blighted body and unhinged mind of his beloved child; and in augmenting, an hundred fold, the cares, anxieties and solicitude of the parent, thus "bowing down (much earlier than would otherwise be the case) his grey hairs with sorrow to the grave." He is sowing seed which will produce a harvest of sorrows. Better would it be for such children, if they never learned their letters.

This course with the child is wholly unnecessary. There is not the least possible need of it. The child may be one of the brightest men of his age, even though he be very backward and very dull in childhood. This was the case with Sir Isaac Newton in philosophy, Walter Scott in fiction, and Andrew Fuller, in polemics.

I have now under treatment for epileptic convulsions, several children where the disease was induced in the manner above described, and this fact has led me to furnish this brief and hasty sketch for your valuable journal. It is hoped it may do some good by admonishing parents, guardians and teachers against crowding upon the minds of children more than the God of nature designed they should bear.—*S. C. Med. Jour.*

